# Iatrogenic Massive Coronary Artery Dissection During Cardiac Catheterization: A Case Report

**DOI:** 10.7759/cureus.60768

**Published:** 2024-05-21

**Authors:** Hemangi Patel, Nithya Devanathan, Mahi Basra, Mark Vinicky, Alejandro Biglione

**Affiliations:** 1 Internal Medicine, Nova Southeastern University Dr. Kiran C. Patel College of Osteopathic Medicine, Fort Lauderdale, USA; 2 Internal Medicine, Nova Southeastern University Dr. Kiran C. Patel College of Osteopathic Medicine, Davie, USA; 3 Osteopathic Medicine, Nova Southeastern University, Clearwater, USA; 4 Medicine, Nova Southeastern University Dr. Kiran C. Patel College of Osteopathic Medicine, Wellington, USA; 5 Internal Medicine, Wellington Regional Medical Center, Wellington, USA

**Keywords:** diagnostic testing, catheter migration, atypical spontaneous coronary artery dissection, cad: coronary artery disease, cardiac cath

## Abstract

Cardiac catheterization is an invasive procedure done for diagnostic and therapeutic purposes to assess coronary artery disease (CAD) and valvular diseases. Although complications rarely occur, they are possible. Of those complications, iatrogenic coronary artery dissection during a coronary catheterization is infrequent and can be severe. This case report discusses a 59-year-old female presenting to the emergency department for sudden onset chest pain, found to have a non-ST-elevation myocardial infarction (NSTEMI), and underwent a left heart catheterization (LHC). During the LHC, she sustained a coronary artery dissection.

## Introduction

Cardiac catheterization is an invasive procedure performed for diagnostic and therapeutic purposes. It is conducted to assess coronary artery disease (CAD), valvular lesions, and cardiac hemodynamics [1]. Additionally, it is utilized to evaluate and treat patients with chest pain of uncertain origin [1]. Prior to the procedure, a medical history is obtained to identify any contrast allergies, a comprehensive examination is conducted to assess the access site, and blood tests are performed to evaluate hemoglobin, platelets, creatinine, and coagulation profile [1].

The most common complications of iatrogenic coronary artery dissections during cardiac catheterization include access site complications, contrast allergies, and myocardial infarctions [1]. While these complications occur relatively infrequently, typically less than 1%, prompt response is crucial when they do occur to prevent exacerbation of morbidity. Iatrogenic coronary artery dissection during coronary catheterization is rare, with an incidence of less than 0.1% and a mortality rate of less than 1% [2]. The mechanism is hypothesized to result from injury to the arterial wall caused by advancing the catheter or wire, injection of contrast media, balloon dilation, or stenting [3,4].

This case involves a 59-year-old female who presented to the Emergency Department with sudden onset chest pain. She was diagnosed with a non-ST-elevation myocardial infarction (NSTEMI) and underwent a left heart catheterization (LHC), during which she sustained a coronary artery dissection. Subsequently, she was immediately transferred to JFK Medical Center.

## Case presentation

A 59-year-old female patient presented to the Wellington Regional Emergency Department after a sudden onset of chest pain. The pain started in the center of her chest and radiated toward the left lateral chest. She began to experience tingling sensations in her upper extremities, along with a sudden onset of weakness and tightness in her chest. Associated symptoms included nausea and vomiting during the onset of chest pain. She took her blood pressure at home when the chest pain started, and it was 232/127 mmHg. She denied any vision changes, slurred speech, headache, or any other symptoms. Her past medical history was significant for hypertension, hyperlipidemia, nine previous transient ischemic attacks (TIAs), vertebral artery dissection, and overactive bladder.

Her past surgical history was significant for a hysterectomy and laser-assisted in situ keratomileusis (LASIK). Her home medications included amlodipine, aspirin, estradiol topical, lisinopril, meloxicam, and zolpidem. She denied alcohol and recreational drug use but admitted to recently quitting smoking after 20 years, although she continues to vape occasionally.

Her vitals on admission were a temperature of 98.4ºF (36.8ºC), a heart rate of 110 beats per minute (BPM), a blood pressure of 196/123 mmHg, a respiration rate of 18 breaths per minute, and an oxygen saturation of 100% in room air. Her labs on admission are shown in Table 1.

**Table 1:** Lab values (normal range) and patient values *Troponin measurement repeated due to initial high reading of 272. Repeated at the two- and four-hour mark to confirm cardiac presentation. ALT, alanine aminotransferase; AST, aspartate aminotransferase; BUN, blood urea nitrogen; HLD, high-density lipoproteins

Test parameter	Normal range	Patient value
Sodium (Na)	135-145 mmol/L	141 mmol/L
Potassium (K)	3.4-4.5 mmol/L	3.5 mmol/L
Chloride (Cl)	95-108 mmol/L	110 mmol/L
Carbon Dioxide (CO_2_)	23-28 mEq/L	24 mEq/L
BUN	8-21 mg/dL	21 mg/dL
Creatinine	0.8-1.3 mg/dL	0.82 mg/dL
AST	5-30 U/L	14 U/L
ALT	5-30 U/L	24 U/L
Alkaline phosphatase	50-100 U/L	62 U/L
Bilirubin	0.3-1.2 mg/dL	0.7 mg/dL
White blood cells	4.5-11 × 10^3^ cells/mcL	7.43 12.69 × 10^3^/mcL
Red blood cells	4.2-5.9 × 10^6^ cells/mcL	4.02 × 10^6^/mcL
Hemoglobin	12-16 g/dL	12.5 g/dL
Hematocrit	36-46%	35.8 %
Platelets	150 ×10^3^ cells/mcL - 450 × 10^3^ cells/mcL	225 × 10^3^ cells/mcL
Lipase	0-160 IU/L	5,221 IU/L
Cholesterol	<200 mg/dL	204 mg/dL
Triglycerides	<150 mg/dL	79 mg/dL
HDL	>40 mg/dL	90 204 mg/dL
Calcium	8.6-10.3 mg/dL	9.6 mg/dL
Troponin	0-0.04 ng/L	272 ng/L *Two hours after admission: 3,342.3 ng/L *Four hours after admission: 3,438.1 ng/L

**Figure 1:**
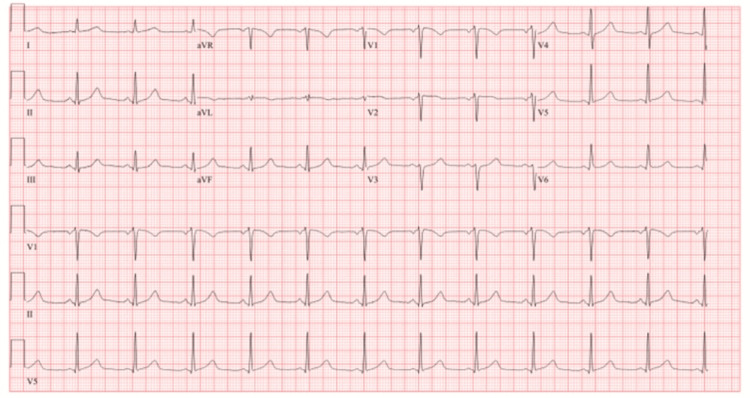
EKG: 72 beats per minute, normal sinus rhythm, and poor R wave progression V1, V2, and V3.

**Figure 2:**
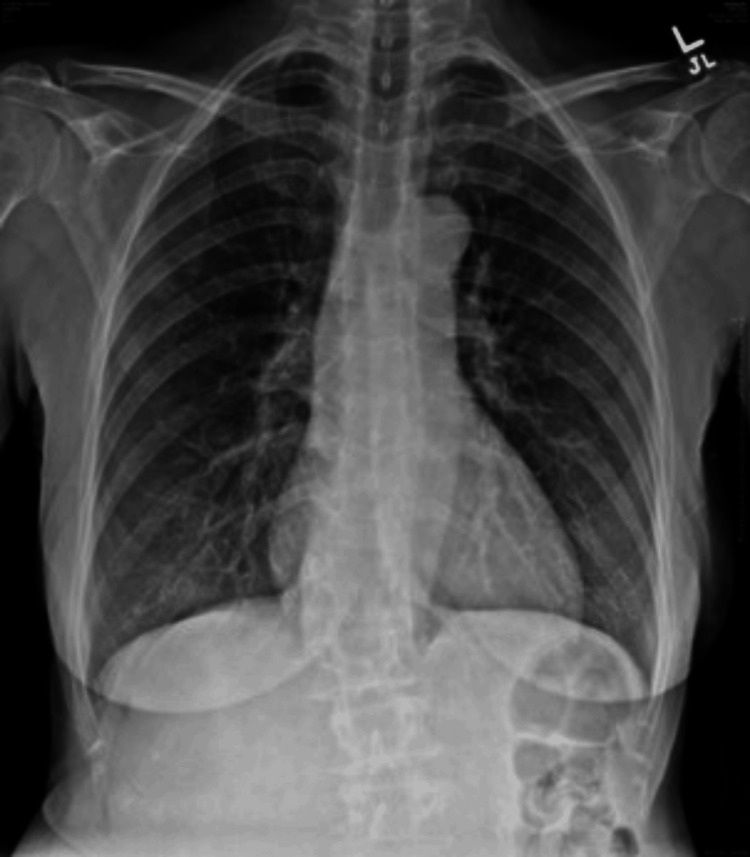
CXR: Cardiomediastinal contours within normal limits, with no infiltrate, effusion, or pneumothorax.

**Figure 3:**
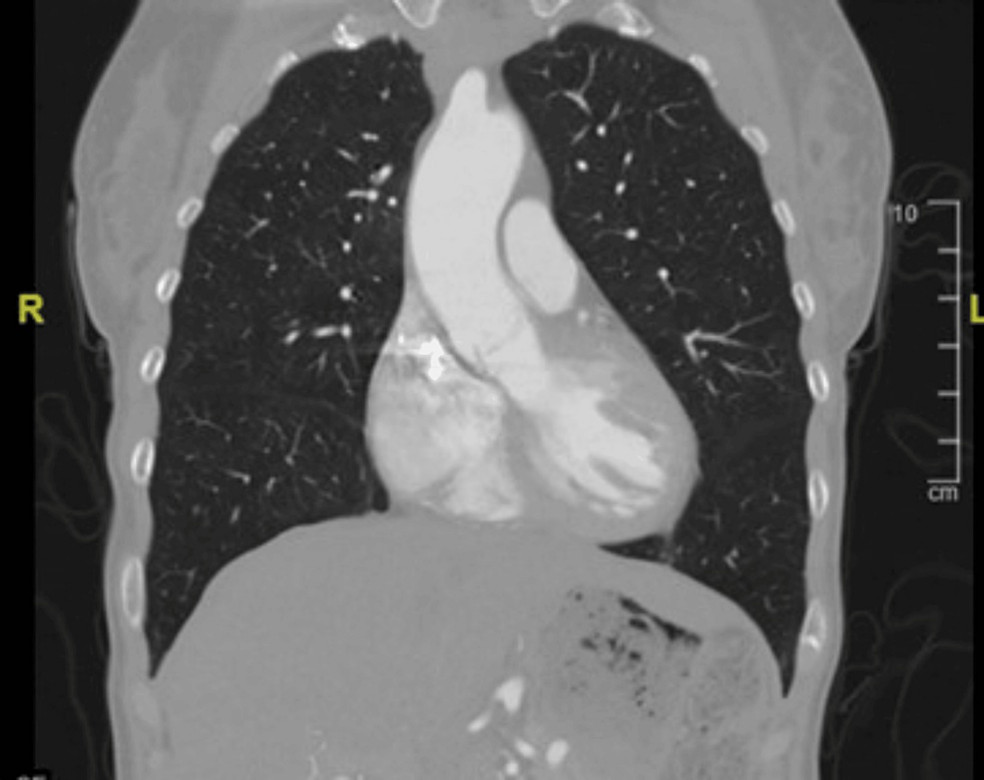
CT angiogram of the chest with contrast: No evidence of pulmonary emboli, aortic aneurysm, or dissection. Dense calcifications are present in the coronary arteries.

The ECG revealed 72 BPM, normal sinus rhythm, and poor R wave progression V1, V2, and V3 (Figure 1). A chest X-ray (Figure 2) revealed cardiomediastinal contours within normal limits, with no infiltration, effusion, or pneumothorax. Then, a CT angiogram of the chest with contrast (Figure 3) was completed and showed no evidence of pulmonary emboli, aortic aneurysm, or dissection, but there were dense calcifications present in the coronary arteries. The lungs were clear with no etiology of an embolism or pleural effusions.

The patient was promptly admitted for treatment of an NSTEMI after receiving Nicardipine 25 mg IV drip for elevated blood pressure and Morphine 4 mg IV push in the emergency room. Throughout her admission, she received treatment with aspirin, amlodipine, atorvastatin, lisinopril, pantoprazole, potassium chloride, heparin drip, and nitroglycerin 1-inch ointment. The cardiology team was consulted upon admission, and an echocardiogram and LHC were recommended.

**Figure 4:**
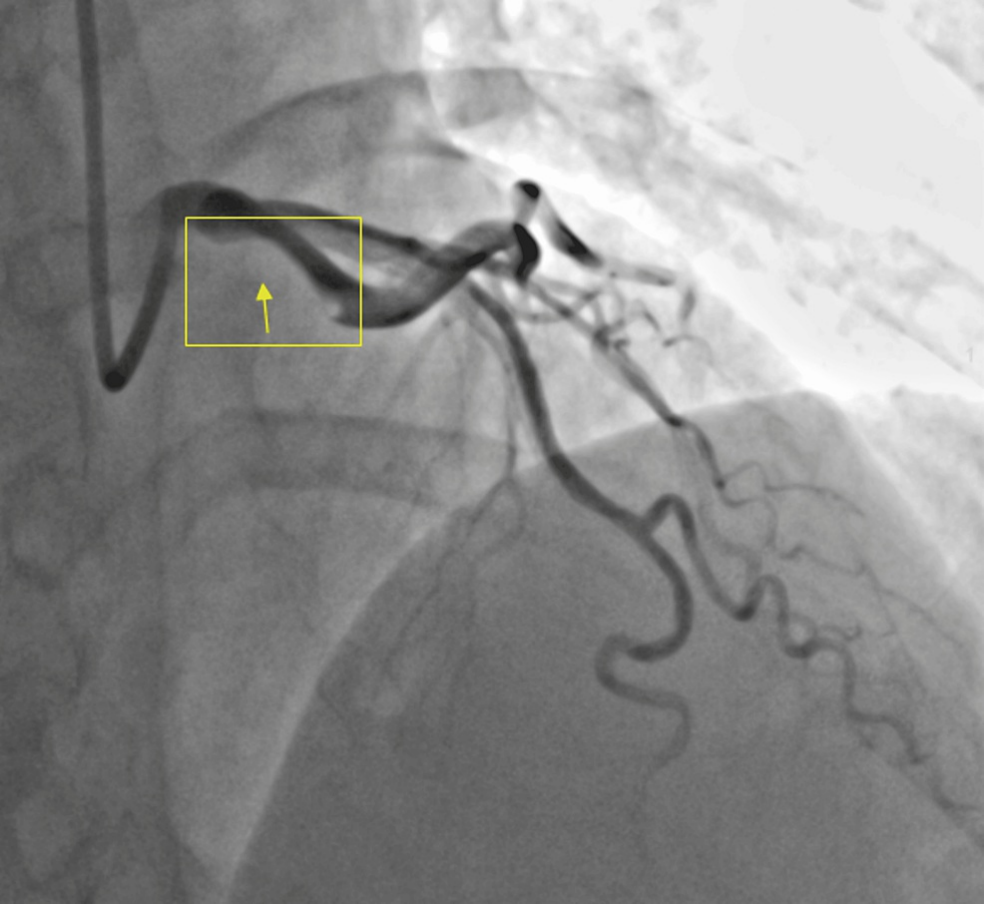
An occlusion of the distal left anterior descending artery (LAD) is visualized in the yellow box, with the yellow arrow pointing to the area of occlusion. The area of the normal artery is seen in black.

An echocardiogram revealed a left ventricular ejection fraction of 45% (standard: 50-70%). The left ventricular wall thickness was normal. The apical anterior, apical septal, apical inferior, apical lateral, and apex left ventricular wall segments were hypokinetic. Otherwise, the remaining wall segments had no abnormalities. The interventricular septal thickness was normal, and the septum was intact. The right ventricle size, thickness, and ventricular systolic function were normal. She was scheduled to undergo an LHC. While undergoing the LHC, an XB 3-5 guiding catheter was placed into the LAD, resulting in the spiraling of the dissection flap into the LAD and circumflex (Figure 4). The catheter was urgently removed, and an intra-aortic balloon pump was placed in her right femoral artery. The cardiac catheterization prior to the insertion of the guide wire revealed a subtotal occlusion of the distal LAD and the right coronary artery with moderate luminal irregularities. Despite experiencing mild chest discomfort during the LHC, there were no EKG changes observed. She was hypertensive and treated with nitroglycerin 50 mg IV and heparin 25,000 units IV to prevent clot formation. An immediate discussion was held with the cardiothoracic surgeon regarding the transfer of the patient to JFK Medical Center for potential revascularization with bypass surgery. The patient was transferred due to the lack of cardiopulmonary bypass ability at Wellington Regional Hospital.

## Discussion

Arterial access is established through the left or the right common femoral or radial artery during the cardiac catheterization procedure. Local analgesics are injected, and the artery is punctured. The needle is advanced into the artery. Anticoagulants, such as heparin, are injected via peripheral venous access to prevent complications. After identifying anatomical landmarks, the femoral artery is punctured using the modified Seldinger method, which involves advancing a 6 French cardiac catheter over a previously used needle and then removing the needle. The fine catheter can then be used to insert the guide wire. Once the guide wire is introduced, an appropriately sized sheath is advanced. Guide catheters are advanced over coronary wires for percutaneous coronary intervention (PCI). Once the appropriate coronary artery is engaged, a coronary guide wire is advanced further, crossing the stenotic area. The lesion is either pre-dilated with a balloon or directly stented [1].

Coronary artery dissection occurs when the arterial wall of the coronary arteries separates due to trauma, spontaneous, or iatrogenic causes [5]. An iatrogenic cause of coronary artery dissection is catheter-induced coronary artery dissection (CICAD), which is extremely rare [6]. There have been fewer than 0.1% of reported CICADs. Those at higher risk include females, patients with preexisting atherosclerotic burden, tortuous arteries, and traumatic sheath placement during the procedure [3,7]. Other predisposing factors include fibromuscular dysplasia, those who are pregnant, early postpartum status, multiparity, and connective tissue disorders. Precipitating factors may include mechanical stressors such as vomiting, coughing, Valsalva-type movements, and emotional stress [8]. The use of large-caliber catheters and poor manipulation of the coronary guidewires used during the procedure also lead to a higher risk of a CICAD [7].

In a CICAD, the mechanism of injury involves disruption to the endothelial cell layer of the coronary artery and the movement of blood into the subendothelial layers of the artery. The blood can then remain localized or flow in an anterograde or retrograde motion [7]. Some important factors to consider in the prognosis of a CICAD are the size of the dissection and antegrade blood flow [7]. In cases with flow-limiting large dissections, where there is a dissection with deterioration of normal blood flow, complications can cause acute limb ischemia and require immediate angioplasty [6]. In most cases, the treatment of choice is stent placement to avoid further complications previously described [3,6,7].

There has been a dramatic increase in PCI being performed in facilities without on-site cardio thoracic surgery. Key structural elements, such as equipment, staffing, supplies, operator requirements, case selection, and surgical consultation, have to meet the standard. An algorithm has been developed to help operators determine the type of patients, lesions, and procedure locations for PCI. The expert consensus typically advises against PCI with limited surgical function in operators who have had less than three years of experience with limited exposure to atherectomy devices and those who have had prior ST elevation or shock [9]. The patient’s adverse outcome may have been due to her risk factors such as being a prior smoker, female, hyperlipidemia, hypertension, and prior history of an artery dissection [3,7-9].

During the LHC of the patient, the procedure resulted in an occlusion of the distal LAD. The guiding catheter was placed into the LAD, resulting in spiraling of the dissection flap into the LAD and circumflex. The catheter was removed, and an intra-aortic balloon pump was placed via the right femoral artery. The reason why this occurred is not known; however, ensuring proper training and expertise, using appropriate catheter sizes and shapes, minimizing catheter manipulation, and monitoring patients closely during and after the procedure for any signs of complications can prevent any life-threatening outcomes in patients undergoing cardiac catheterizations.

## Conclusions

Cardiac catheterization is a procedure involving catheter insertion for both diagnostic and therapeutic reasons to evaluate CAD and valvular conditions. CICAD is a rare complication of catheter placement with a high mortality rate in affected patients. Mechanisms of coronary arterial dissections during the procedure are hypothesized to be caused by injury to the arterial wall during catheter or wire manipulation, contrast injection, balloon dilation, or stenting. Catheters should be manipulated cautiously, and injection should only be done when the catheter is safely secured. If a dissection occurs, appropriate intervention must be done emergently to improve survival outcomes. This case report highlights the necessity for physicians to be attentive during cardiac catheterizations to avoid complications in patients.
